# Differential mRNA and long noncoding RNA expression profiles in pediatric B-cell acute lymphoblastic leukemia patients

**DOI:** 10.1186/s12887-021-03073-5

**Published:** 2022-01-03

**Authors:** Jing Xia, Mengjie Wang, Yi Zhu, Chaozhi Bu, Tianyu Li

**Affiliations:** 1 Department of Pediatric Laboratory, The Affiliated Wuxi Children’s Hospital of Nanjing Medical University, Wuxi, 214000 Jiangsu China; 2 Department of hematology & oncology, The Affiliated Wuxi Children’s Hospital of Nanjing Medical University, Wuxi, 214000 Jiangsu China; 3 Center of Reproductive Medicine, State Key Laboratory of Reproductive Medicine, Research Institute for Reproductive Health and Genetic Diseases, The Affiliated Wuxi Matemity and Child Health Care Hospital of Nanjing Medical University, Wuxi, 214002 Jiangsu China

**Keywords:** Acute lymphoblastic leukemia, Long non-coding RNA, mRNA, Next generation sequencing, Transcriptome network

## Abstract

**Background:**

Long non-coding RNAs (lncRNAs) are transcripts longer than 200 nucleotides (nt) that are involved in the pathogenesis and development of various cancers including B cell acute lymphoblastic leukemia (B–ALL). To determine the potential roles of lncRNAs involved in pathogenesis of B-ALL, we analyzed the expression profile of lncRNAs and mRNAs in B-ALL, respectively, and constructed lncRNAs/mRNAs interaction network.

**Methods:**

We performed RNA sequencing of 10 non-leukemic blood disease donors and 10 B-ALL patients for Gene Ontology (GO) and the Kyoto Encyclopedia of Genes and Genomes (KEGG) analysis. Interactions among mRNAs were predicted using the STRING database. Quantitative real time PCR (qRT-PCR) was performed to verify the RNA-seq data of lncRNAs and mRNAs. Potential functions of subtype-specific lncRNAs were determined by using coexpression-based analysis on distally (trans-pattern) located protein-coding genes.

**Results:**

A total of 1813 differentially expressed transcripts (DETs) and 2203 lncRNAs were identified. Moreover, 10 dysregulated lncRNAs and 10 mRNAs were randomly selected, and further assessed by RT-qPCR in vitro. Go and KEGG analysis demonstrated that the differentially expressed mRNAs were most closely associated with myeloid leukocyte activation and in transcriptional misregulation in cancer, respectively. In addition, co-expression analysis demonstrated that these lncRNAs, including MSTRG.27994.3, MSTRG.21740.1, ENST00000456341, MSTRG.14224.1 and MSTRG.20153.1, may mediate the pathogenesis and development of B-ALL via lncRNA-mRNA network interactions.

**Conclusions:**

These results showed that several mRNAs and lncRNAs are aberrantly expressed in the bone marrow of B-ALL patients and play potential roles in B-ALL development, and be useful for diagnostic and/or prognostic purposes in pediatric B–ALL.

**Data availability:**

The datasets used during our study are available through HARVARD Dataverse Persistent ID doi:10.7910/DVN/LK9T4Z.

**Supplementary Information:**

The online version contains supplementary material available at 10.1186/s12887-021-03073-5.

## Background

Acute lymphoblastic leukemia (ALL) is the most common malignancy in children, accounting for approximately 30% of all childhood cancers and approximately 80% of all childhood leukemia. Of these incidences of childhood leukemia, 80% are derived from B-cell progenitors (B-ALL) [1]. B-ALL has one of the highest cure rates of pediatric malignancies [2, 3]. However, although most patients can be cured, higher performance therapies for ALL are required to improve the unfavorable outcomes. Thus, it is necessary to achieve an improved understanding of its potential pathogenesis.

Transcriptome profile analysis is an advanced method that has been applied to reveal the etiology underlying the specific biological processes of ALL. It benefits from ribose nucleic acid sequencing (RNA-seq), which is the most extensively used technology for detecting transcriptomes [4]. Previous studies comparing the pathophysiologies of ALL to chronic lymphocytic leukemia (CLL) or comparing the molecular pathogenesis features of adult and pediatric ALL, have focused on analyzing the expression profiles of microRNAs (miRNAs), messenger RNA (mRNA), and miRNA/mRNA networks [5–7]. Long non-coding RNAs (lncRNAs), which are one of the most studied non-coding RNA types, are over 200 nucleotides in length. LncRNAs can act as gene expression modulators at the epigenetic, transcriptional, and post-transcriptional levels. While the roles of lncRNAs in ALL and their underlying mechanisms have been continuously investigated, related data of research articles remain scarce. Fernando et al. demonstrated that the lncRNA CASC15 is fundamental for the cellular survival and proliferation of ALL cells, as it regulates SOX4 expression [8]. Another study has suggested that the lncRNA BALR-6 may have an effect on proliferation and apoptosis in ALL cell lines [9]. In addition, in vitro studies have shown that the lncRNA MALAT1 affects ALL cell proliferation and apoptosis via regulating the miR-205-PTK7 axis. Furthermore, the lncRNA CRNDE has been shown to upregulate CREB expression by suppressing miR-345-5p, promoting cell proliferation, and reducing cell apoptosis in ALL [10, 11]. All of these above studies indicate that lncRNAs could affect the expression of mRNAs via co-expression networks. Nevertheless, the role of lncRNA-mRNA networks in the pathogenesis of B-ALL is not fully understood. This study analyzed the expression profiles of lncRNAs and mRNAs in B-ALL, compared with non-leukemic blood disease controls, by using Next Generation Sequencing (NGS). Specific focus was given to differential mRNA and lncRNA expression levels and lncRNA-mRNA networks to identify the potential underlying mechanism of B-ALL. This approach may provide novel insights into further functional studies, and may reveal new biomarkers for the diagnosis of B-ALL.

## Materials and methods

### Patients and samples

Bone marrow samples were obtained at diagnosis from ten pediatric B-ALL patients. Further samples from ten pediatric donors with common blood diseases (CBDs), diagnosed as anemia and agranulocytosis; these were used as controls to lessen the potential variation. All samples were collected from patients at the Affiliated Wuxi Children’s Hospital of Nanjing Medical University. The pediatric B-ALL patients included in present study were diagnosed in accordance with the revised French-American-British classification and by analysis of leukemic cells with respect to morphology, immunophenotype, and cytogenetics. Bone marrow samples were submitted to cell separation using Ficoll-Paque (GE Healthcare, USA) and the isolated mononuclear cell fraction was used for the subsequent extraction of the total RNA. The study protocol was approved by the institutional review Board of the Affiliated Wuxi Children’s Hospital of Nanjing Medical University (WXCH 2020–08-004). This trail was conducted basing on the written informed consent of all patients, and particularized information is summarized in Supplementary Table S1.

### RNA extraction, strand-specific library construction and sequencing

Total RNA from mononuclear cells was extracted using a Trizol reagent kit (Invitrogen, Carlsbad, CA, USA) consistent with the manufacturer’s instructions. RNA quality was appraised using an Agilent 2100 Bioanalyzer (Agilent Technologies, Palo Alto, CA, USA). After total RNA was extracted, rRNAs were removed to retain mRNAs and non-coding RNAs (ncRNAs). The enriched mRNAs and lncRNAs were fragmented into short pieces using fragmentation buffer, and were then reverse transcribed into complementary deoxyribose nucleic acid (cDNA) using random primers. Second-strand cDNA were synthesized using DNA polymerase I, RNase H, dNTP (dUTP instead of dTTP), and buffer. Next, the cDNA fragments were purified using a QiaQuick polymerase chain reaction (PCR) extraction kit (Qiagen, Venlo, The Netherlands). They were then end repaired, poly (A) added, and bound to Illumina sequencing adapters. Then, the digested second-strand cDNA using Uracil-N-Glycosylase (UNG) were size selected by programming steps including agarose gel electrophoresis, PCR amplified, and sequenced using Illumina HiSeqTM 4000 (or other platforms) by Gene Denovo Biotechnology Co. (Guangzhou, China). The datasets used during our study are available through HARVARD Dataverse Persistent ID doi:10.7910/DVN/LK9T4Z.

### Filtering of clean reads

All following data processing and analysis were performed according to the schematic illustration in Supplementary Fig. S1. Reads obtained from the sequencing machines included raw reads containing adapters or, which could have affected the following assembly and analysis. To procure high quality clean reads, raw reads including low quality bases or adapters that may interfere subsequent analysis were filtered using fastp (version 0.18.0). The parameters were as follows: 1) removing reads containing adapters, 2) removing reads containing more than 10% of unknown nucleotides (N), and 3) removing low quality reads containing more than 50% of low quality (Q-value≤20) bases.

### Differentially expressed transcripts (DETs) analysis

The DETs of coding RNAs and lncRNAs were analyzed respectively. DESeq2 software was used to analyze differential expression of mRNAs and lncRNAs between two different groups (CBD group versus B-ALL group). Those transcripts with the parameters of a false discovery rate (FDR) of < 0.05 and an absolute fold change (FC) of ≥2 were considered to be differentially expressed. Enrichment analysis of Gene Ontology (GO) functions, Kyoto Encyclopedia of Genes (KEGG) pathways, Disease Ontology (DO), Reactome Enrichment Analysis, and Gene Set Enrichment Analysis (GESA) were performed by choosing the differentially expressed coding RNAs.

### Functional enrichment analysis

GO enrichment analysis was performed basing on three terms including biological processes, cellular components and molecular functions (http://www.geneontology.org/). KEGG enrichment analysis distinguished significantly enriched biological pathways. DO enrichment analysis identified significantly enriched human disease DO terms in DETs. Reactome enrichment analysis identified significantly enriched reactions including signaling, innate and acquired immune function, transcriptional regulation, translation, apoptosis, and classical intermediary metabolism in DETs. GESA analysis was performed using the software packages GSEA and MSigDB to identify genes in specific GO terms\pathways\DO terms showing significant differences between the two groups. All above analyses meeting the condition of *p* < 0.05 were considered to be significant.

### LncRNA-mRNA association analysis

The lncRNA-mRNA association analysis was based on lncRNA trans-regulation analysis to reveal the interaction between lncRNA and mRNA and identify target genes of lncRNAs.

### Validation of RNA-seq results

A number of mRNAs and lncRNAs between the paired groups were selected to validate the RNA-seq data by quantitative real time PCR (qRT-PCR). After determining the best annealing temperatures, qRT-PCR was performed on the ABI 7500 system (Applied Biosystems, Foster City, CA,USA) to detect the relative expression levels of mRNA and lncRNA using SYBR Premix ExTaq II (DRR820; TaKaRa). The relative expression levels of each mRNA/lncRNA were calculated using comparative cycle threshold method (∆∆Ct). Standardization was performed based on the internal reference of GAPDH. All data are presented herein as the average of three independent experiments.

### Statistical analysis

Hypergeometric Test was used to calculate the enrichment of GO terms, KEGG pathways, and DO enrichment analysis to show the significance of enrolled transcripts. Quantitative data was assessed with the use of two independent samples t-test. Data are presented herein as the mean ± standard deviation. The edgeR software package (version 3.28.1) was used to identify differentially expressed lncRNAs and mRNAs between paired groups. *p* < 0.05 was considered to indicate a statically significant difference.

## Results

### Differentially expressed lncRNAs and mRNAs in B-ALL

Coding Potential Calculator (CPC) 2 and Coding-Non-Coding Index (CNCI), two efficient tools for distinguish protein-coding and non-coding sequences, were used to perform prediction analysis of the coding capabilities of novel lncRNAs. The number of non-coding transcripts were 8049 and 7289 following CPC2 and CNCI analysis, respectively (Fig. S2A). The overlaps of novel 6283 non-coding lncRNAs were enrolled for subsequent expression profile analysis combined with the known lncRNAs. According to the position relation of lncRNAs and adjacen mRNAs, lncRNAs were classified into six categories: 47.0% were intergenic, 11.2% were antisense, 32.6% were sense, 3.0% were intronic, 4.4% were bidirectional, and 1.8% were others (Fig. S2B).

**Fig. 1:**
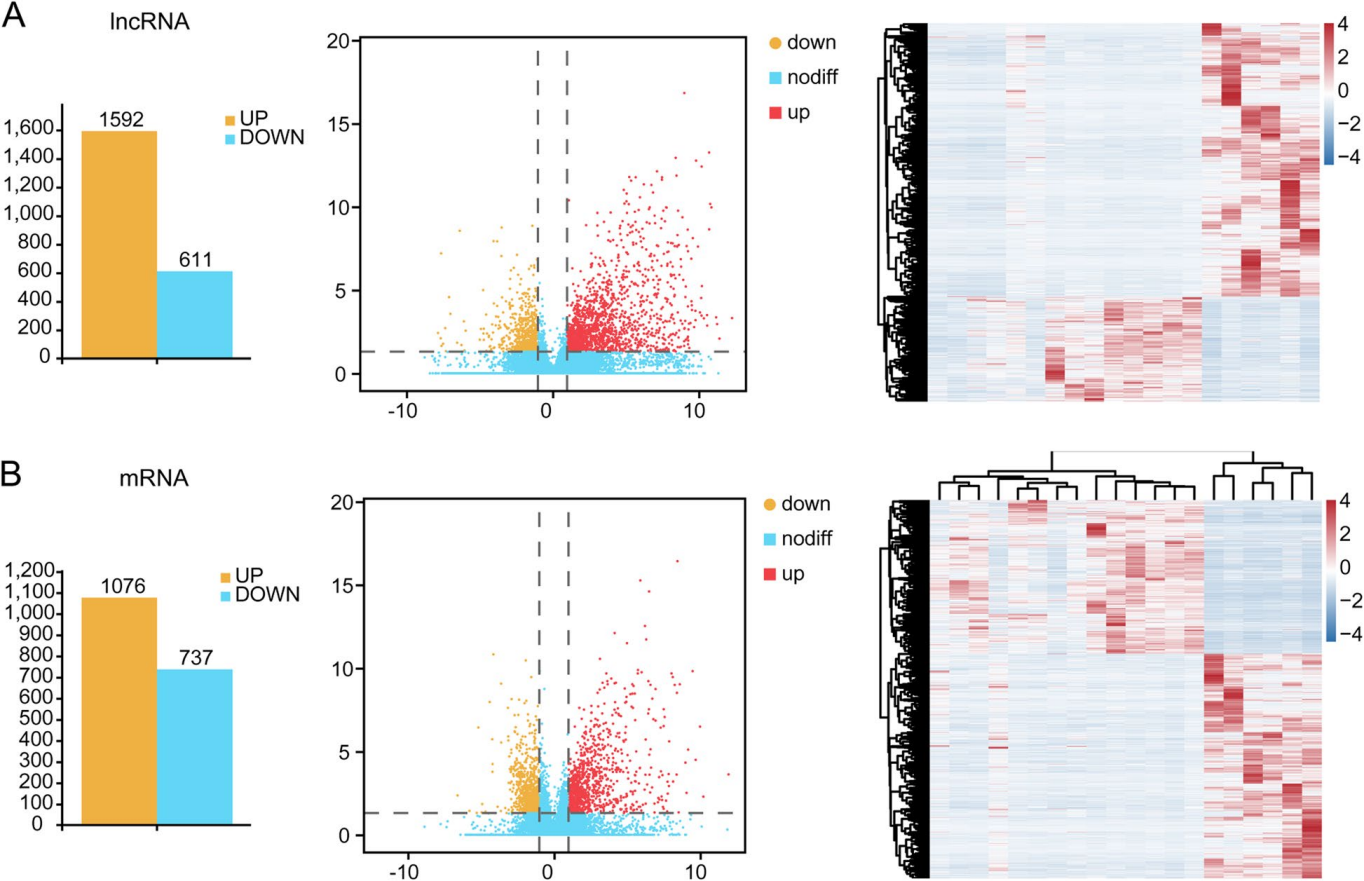
Histogram, volcano plots and heat map showing expression profiles of lncRNAs (**A**) and mRNAs (**B**) in B-ALL. Left panels, histogram visualizing the differentially expressed lncRNAs and mRNAs. Middle and right panels, volcano plots and heat maps showing significantly changed lncRNAs and mRNAs with fold change≥2.0, respectively (*p* < 0.05; FDR < 0.05)

Histogram, volcano plots and heat maps were used for assessing gene expression variation between CBD and B-ALL groups. In total, 2203 lncRNAs displayed differential expression in B-ALL, including 1592 upregulated lncRNAs and 611 downregulated lncRNAs (Fig. 1A). Of 1813 mRNAs that showed differential expression, 1076 were upregulated and 737 were downregulated (Fig. 1B). Among them, 376 lncRNAs and 105 mRNAs were significantly upregulated, and 15 lncRNAs and 4 mRNAs were significantly downregulated > 5(Log_2_FC) in B-ALL. In-depth analysis showed that 16 lncRNAs and 2 mRNAs (IGKV1OR2–108 and IRX2) were significantly upregulated > 10(Log_2_FC) in B-ALL. Hierarchical clustering analysis showed systematic variations in the expression of lncRNAs and mRNAs among samples. The data suggested that the expression of lncRNAs and mRNAs in B-ALL differ from those in CBD group. All differentially expressed lncRNAs are listed in Supplementary Table S2 and differentially mRNAs expressed are listed in Supplementary Table S3.

### Validation of differentially expressed lncRNAs and differentially expressed mRNAs using qPCR in B-ALL

**Fig. 2:**
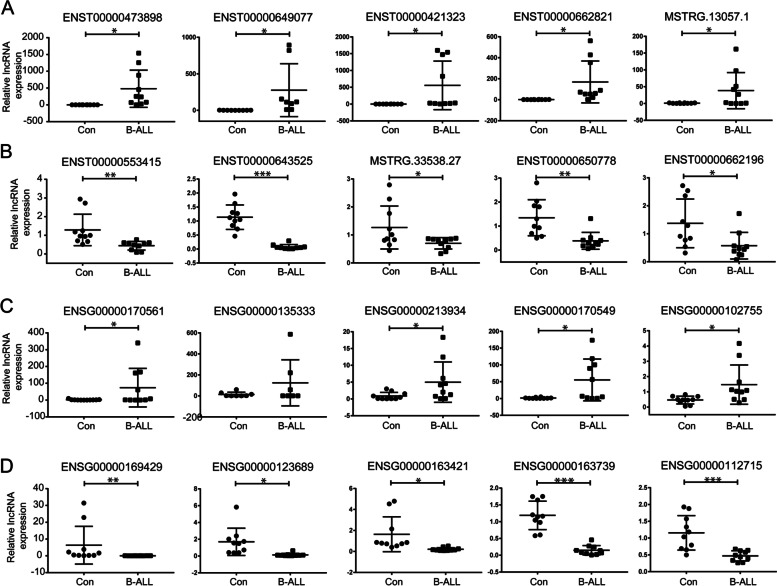
The dysregulated lncRNAs and mRNAs by qPCR in vitro in B-ALL. **A** Validation of upregulated lncRNAs; **B** Validation of downregulated lncRNAs; **C** Validation of upregulated mRNAs; **D** Validation of downregulated mRNAs. *p < 0.05, **p < 0.01, and ***p < 0.001

To validate the RNA-seq data accuracy, we randomly selected 10 lncRNAs and 10 mRNAs to assess the RNA-seq results by qRT-PCR using PCR primers as documented in Supplementary Table S4. The present results indicated that the lncRNA expression levels of ENST00000473898, ENST00000649077, ENST00000421323, ENST00000662821 and MSTRG.13057.1 were upregulated in B-ALL compared to CBD (Fig. 2A), whereas the expression levels of ENST00000553415, ENST00000643525, MSTRG.33538.27, ENST00000650778 and ENST00000662196 in B-ALL were downregulated (Fig. 2B). Moreover, comparing B-ALL with CBD, the mRNA expression levels of ENSG00000170561 (IRX2), ENSG00000135333 (EPHA7), ENSG00000213934 (HBG1), ENSG00000170549 (IRX1) and ENSG00000102755 (FLT1) were upregulated (Fig. 2C), while ENSG00000169429 (CXCL8), ENSG00000123689 (GOS2), ENSG00000163421 (PROK2), ENSG00000163739 (CXCL1), ENSG00000112715 (VEGFA) in B-ALL were downregulated (Fig. 2D). Therefore, the qPCR results were conclusively coincided with those identified in RNA-seq data.

### Enrichment analysis of differentially expressed genes

**Fig. 3:**
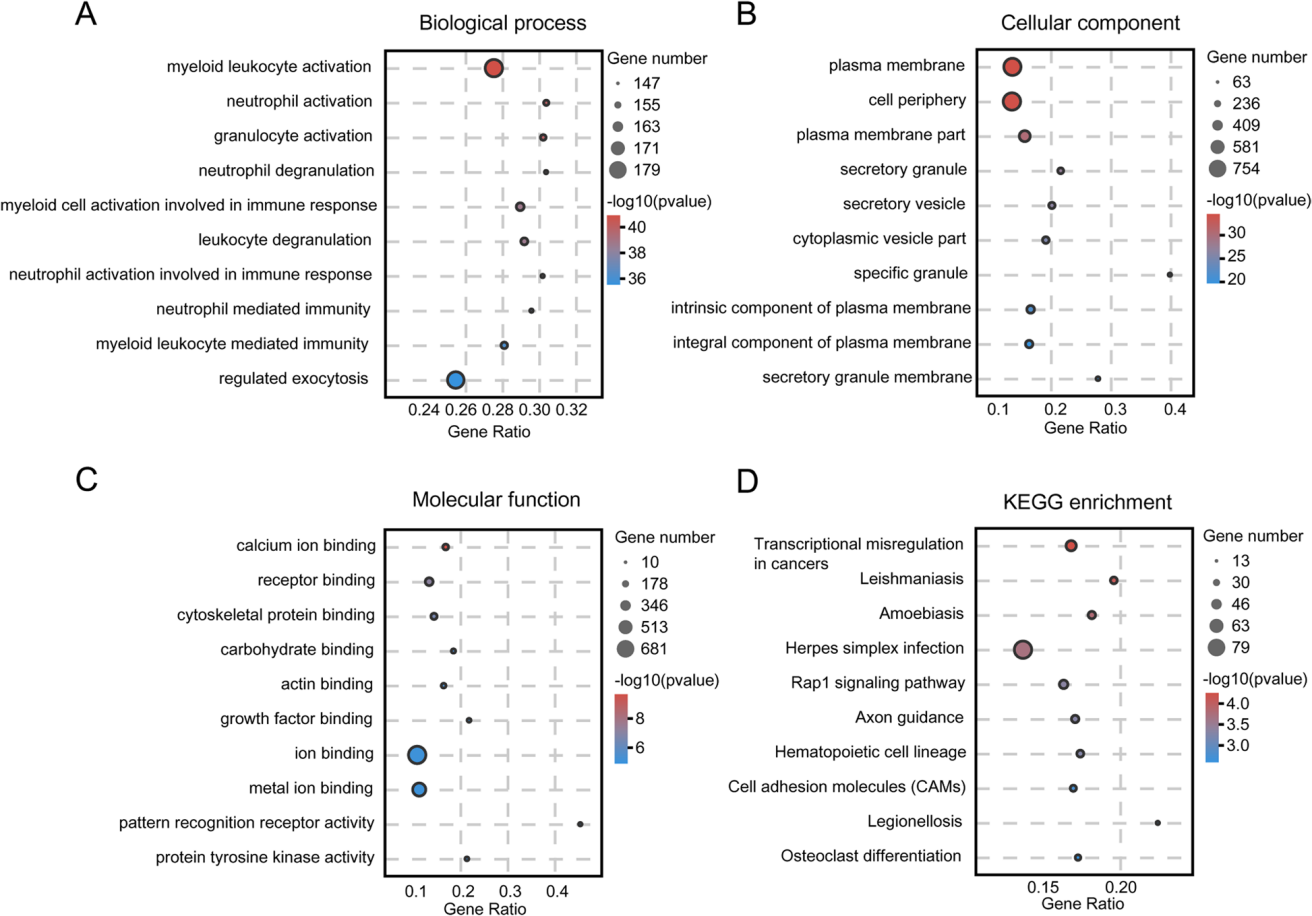
Gene ontology and pathway analysis predicting the potential function of the differentially expressed mRNAs with top 10 enrichment scores in B-ALL. **A** Biological process category. **B** Cellular component category. **C** Molecular function category. **D** Canonical signaling pathways

The GO and KEGG pathway analyses of differentially expressed mRNAs were performed to provide a clue about the B-ALL disease process, which may also reveal the role of lncRNAs in B-ALL. We enrolled all differentially expressed mRNAs for the GO analysis and found that the most enriched GO terms by upregulated and downregulated transcripts were most highly involved in myeloid leukocyte activation, plasma membrane and calcium ion binding in corresponding biological process category (Fig. 3A), cellular component category (Fig. 3B) and molecular function category, respectively (Fig. 3C). KEGG pathway analysis results demonstrated that the differentially expressed mRNAs were mainly enriched in transcriptional misregulation in cancer, leishmaniasis, amoebiasis, herpes simplex infection and Rap1 signaling pathway (Fig. 3D).

Disease Ontology (DO) analysis demonstrated that highly enriched disease were lymphoblastic leukemia, arthritis and bone inflammation disease, which were associated with the etiology or clinical symptom of B-ALL (Fig. S3A). In addition, the Reactome considered as an improved pathway database was used for Entities (nucleic acids, proteins, complexes and small molecules) annotation of enriched pathways. Results showed that differentially expressed mRNAs were involved in neutrophil degranulation, innate immune system and exocytosis of specific granule lumen proteins in B-ALL (Fig. S3B).

### Functional analyses of the mRNAs

**Fig. 4:**
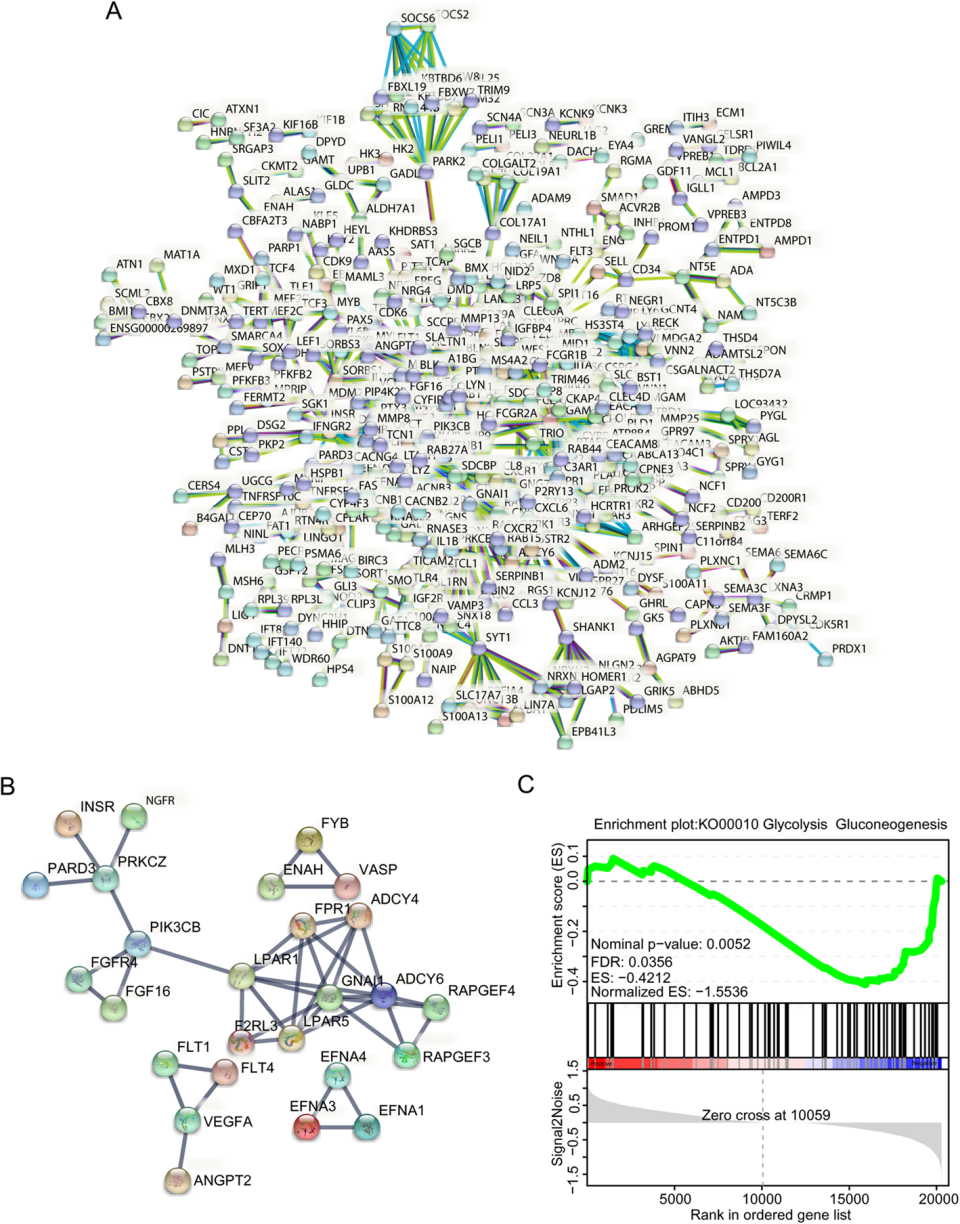
Protein-protein interaction networks by STRING and GSEA analysis of enriched pathway. **A** STRING was used to construct the DETs (FC ≥ 2, *p* < 0.05, FDR < 0.01) network and confidence score was set as the highest (> 0.9). **B** Subnetwork of all DETs enriched in Rap1 signaling pathway. **C** GSEA analysis of Starch and sucrose metabolism pathway

To further explore the function of genes at the protein level, and to reveal the core mRNAs in the cellular process of B-ALL, Search Tool for the Retrieval of Interacting Genes/Proteins (STRING) was used to filter functional genes constructing an interaction network for annotating structural and functional properties of proteins [12]. Considering that too many proteins show interactive function, the threshold of confidence score > 0.9 and FDR < 0.01 were adopted to assess the interactive strength of protein-protein encoded by the DETs. The network contains 1191 nodes and 2014 edges with protein–protein interaction enrichment *p*-value of 1.0e-16 (Fig. 4A). The highest scores were 0.99 among which protein pairs of VEGFA and FLT1 drew our attention in Rap1 signaling pathway that was one of top 5 enriched pathway by KEGG analysis due to the two encoding genes coincidently differentially expressed in Rap1 signaling pathway. It was demonstrated that whether Rap1 activation was involved in the notch-dependent leukemogenicity of T-ALL [13, 14]. The networks enriched in Rap1 signaling pathway were performed with STRING (confidence score > 0.9, FDR < 0.01) to illustrate the interaction of proteins in this pathway (Fig. 4B). Although another enriched pathway of Glycolysis/Gluconeogenesis did not conform to the threshold of FDR < 0.01, DETs including ENO1, a key enzyme functioning in glycolysis process were enriched in Glycolysis/Gluconeogenesis pathway following the GSEA analysis (NES = -1.553, *p* = 0.005, FDR = 0.036) (Fig. 4C), which indicated that the pathogenesis of B-ALL were associated with the deregulated expression of glycometabolism-related genes resulting in abnormal energy supply.

### LncRNA/mRNA co-expression network and GO/KEGG annotations in B-ALL

**Fig. 5:**
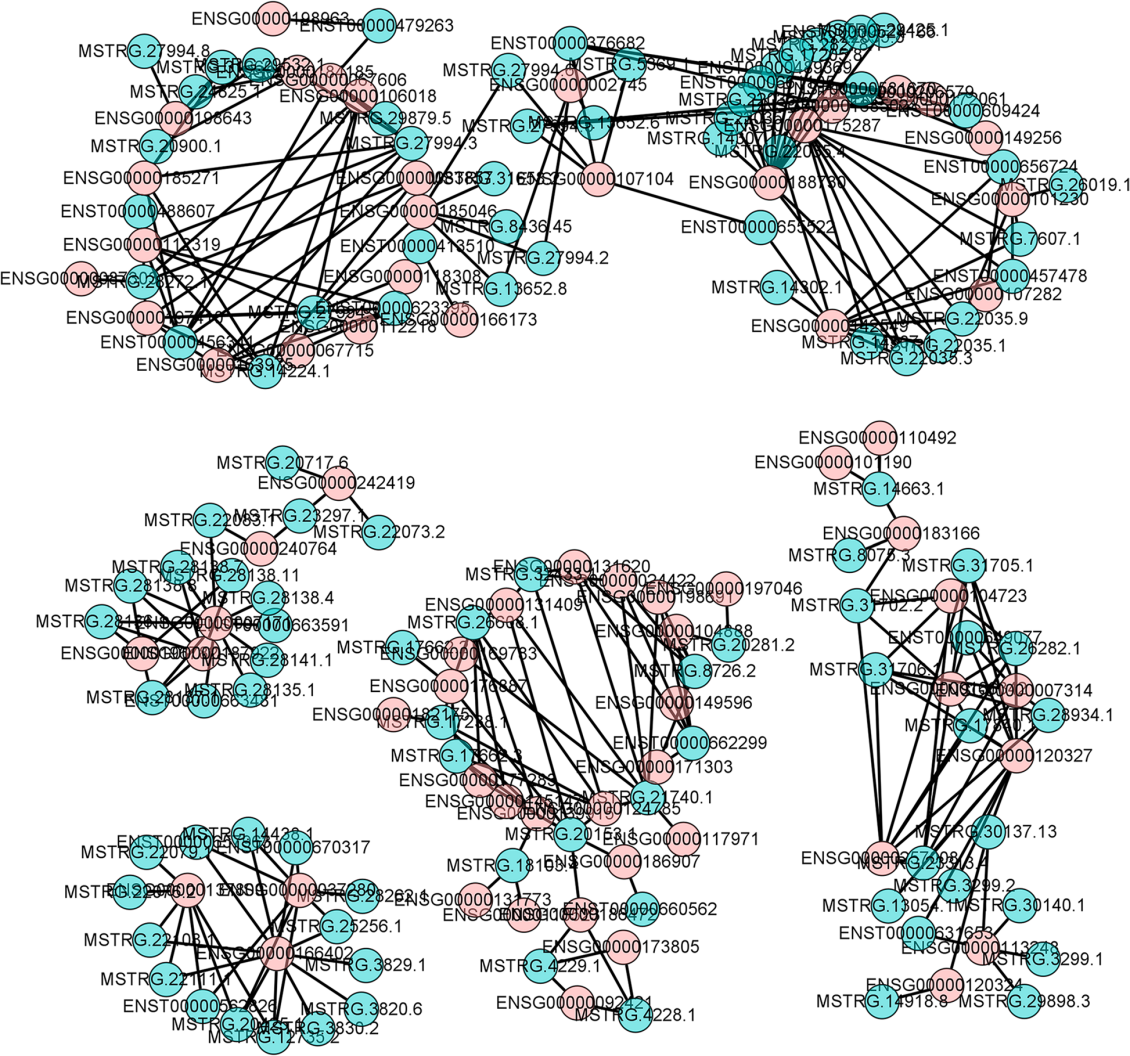
Co-expression networks constructed basing on the Pearson’s correlation (> 0.99). Pink, mRNA; pale green, lncRNA

It is well known that antisense lncRNAs may regulate the gene silencing, transcription and stability of mRNA by binding to its sense strand, and lncRNAs could modulate the expression of neighboring coding genes by cis-pattern [15, 16]. Trans-pattern analysis independent on the position of the encoding gene is able to construct correlation or co-expression network between lncRNA and mRNA. We performed trans-pattern analysis by enrolling the differently expressed lncRNA and mRNA (FC ≥ 2, FDR < 0.01) (Supplementary Table S5), and found that basing on the threshold of Pearson’s correlation (> 0.99), lncRNAs MSTRG.27994.3, MSTRG.21740.1, ENST00000456341, MSTRG.14224.1 and MSTRG.20153.1 seemed to be the vital parts in the network interacting with at least 7 DETs that contained a differently expressed mRNA of PRKCZ that acts in the Rap1 signaling pathway (Fig. 5), among which MSTRG.21740.1 interacted with 8 mRNAs. Therefore, the present results suggested that these lncRNAs may serve important roles in the pathogenesis of B-ALL.

In addition, the enriched GO term was used to examine the potential function of mRNAs related to differentially expressed lncRNAs following trans-pattern analysis. For biological process, the top 3 enriched term were biological adhesion (GO:0022610), homophilic cell adhesion via plasma membrane adhesion molecules (GO:0007156), and cell-cell adhesion via plasma-membrane adhesion molecules (GO:00398742) (Fig. S4A); for cellular component, the top 3 enriched term were plasma membrane part (GO:0044459), cell periphery (GO:0071944), and intrinsic component of plasma membrane (GO:0031226) (Fig. S4B); for molecular function, the top 3 enriched term were calcium ion binding (GO:0005099), transmembrane receptor protein tyrosine kinase activity (GO:0004714), and transmembrane receptor protein kinase activity (GO:0019199) (Fig. S4C). In this study, the majority of mRNAs appear to be associated with immune response, metabolic process, and transcriptional activation. Furthermore, the KEGG results showed that lncRNA/mRNA co-expression analysis by trans-pattern were mainly involved in axon guidance, herpes simplex infection, Rap1 signaling pathway, transcriptional misregulation in cancer and MicroRNAs in cancer (Fig. S4D), which suggested that Rap1 signaling pathway also played an important role in the lncRNA/mRNA network.

## Discussion

Previous studies regarding the transcript results of B-ALL patients have identified a number of lncRNAs and vital protein–coding genes that are associated with the etiology or clinical outcome of B-ALL [17–19]. To the best of the authors’ knowledge, although some studies have performed transcriptome profile analysis to investigate the role of lncRNA in the pathogenesis of B-ALL, the small sample sizes of parallel controls and the restricted numbers of isolated cell types limited the general conclusions of these studies [20–22]. All above related reports were summarized in Supplementary Table S6. Moreover, microarray data prevented the identification of novel transcripts. In the present study, a comprehensive analysis of lncRNA and mRNA profiling data was conducted between B-ALL patients and CBD controls by NGS. Several differently expressed lncRNAs and mRNAs were identified, in which some were validated by qRT-PCR; functional annotations were also carried out. Furthermore, lncRNA-mRNA network analyses were preformed to extract candidate lncRNAs interacting with the aberrantly expressed mRNAs, revealing that five key lncRNAs were co–expressed with at least seven mRNAs in the network, bases on a very high threshold of 0.99 for the Pearson’s correlation. These findings could promote the understanding of the functional mechanism through which lncRNA interacts with mRNA, thus playing an important role in B–ALL pathogenesis.

The biological functions and potential pathways of mRNAs associated with differentially expressed lncRNAs were preliminarily predicted by GO and KEGG pathway analyses. Extraordinarily, significantly enriched pathways were closely associated with signal transduction, metabolic process, infections, and the immune system, which all play important roles in B-ALL development and disease treatment. The KEGG and co-expression analysis results showed that the Rap1 signaling pathway was exclusively highly enriched in B-ALL, when compared to controls. It was demonstrated that the Rap1 signaling pathway is associated with leukemia cell adhesion and migration, suggesting that Rap1 could participate the process of notch activation and leukemogenicity of T-ALL. This suggests that the Rap1 signal may function in the notch-dependent B-ALL development and its progression [14, 23, 24]. Infante et al. reported that Rap1b is a subtype of Rap1, and that its depletion could be used to reduce tissue invasion in T-ALL. Furthermore, another integrative network analysis of pediatric acute lymphoblastic leukemia revealed that gene expression and methylation consistently targeted the Rap1 signaling pathway [25]. In addition, the direct activation of Rap1 can lessen the cellular actions of leptin and correct glucose imbalance in obese mouse, which suggests that Rap1 is associated with energy metabolism [26]. VEGFA and FLT1, two differentially expressed genes, presented the highest correlation score of 0.99 by STRING in the Rap1 signaling pathway. Previous studies have shown that increased levels of Helios Treg cells promoted angiogenesis in the bone marrow of ALL mice via the VEGFA/VEGF receptor 2 pathway [27]. Furthermore, FLT-1 is generally expressed in pediatric ALL and VEGFA/FLT-1 signaling enhances the migration and survival of leukemia [28, 29]. FLT-1 activation on ALL cells results in cell migration and proliferation in vitro. In addition, FLT-1 neutralization affects leukemia localization, increases leukemia apoptosis, and impedes the exit of ALL cells, thus prolonging the survival of inoculated mice. All of the above results indicate that the VEGFA/FLT-1 interaction could play important roles in regulating the development of B-ALL. PRKCZ is a calcium- and diacylglycerol-independent serine/threonine protein kinase that functions in the phosphatidylinositol 3-kinase (PI3K) pathway and the mitogen-activated protein (MAP) kinase cascade. It is also involved in NF-kappa-B activation, mitogenic signaling, cell proliferation, and inflammatory response. AKT, PI3K2CB, and some other genes also feature in the pathway upstream of PRKCZ. The PI3K/Akt and NF-κB signaling pathways have been extensively demonstrated to participate in the regulation of migration, and in the viability of leukemic cells, both in vivo and in vitro [30–32]. It has also been reported that the knockdown of PRKCZ leads to more rapid losses of DNA mismatch repair enzyme (MSH2) proteins, resulting in significant reductions in DNA mismatch repair (MMR) and increased resistance to thiopurines for ALL [33]. Arsenic trioxide (ATO) has been demonstrated to interrupt the function of the PI3K/Akt pathway in ALL; PRKCZ may be responsible for the provocation of resistance to ATO [34]. The most aberrant upregulated lncRNA, ENST00000473898, showed a high correlation score of 0.95 with PRKCZ, suggesting that an in-depth study should be conducted to elucidate its potential regulatory mechanism.

The lncRNA-mRNA network constructed in the present study revealed that some lncRNAs may exert significant effects in B-ALL by regulating downstream mRNAs. Affinito et al. specially constructed a lncRNAs-mRNAs co-expression network in childhood B-ALL, based on previous RNA-sequencing experiments [35]. In said experiments, gene expression profiling using RNA-seq of leukemic cells purified from three pediatric B-ALL patients (compared with that observed in mature B cells from the peripheral blood of three healthy donors) highlighted that 24 key lncRNAs and their co-expressed mRNAs may play important roles in B-ALL pathogenesis [36]. Unlike this study, however, here larger sample sizes of B-ALL patients were used alongside quantity-matched controls. Furthermore, the control group used the same cell type of mononuclear cells derived from bone marrow. Nevertheless, there is a potential limitation for our study. Considering the difficulty in obtaining the healthy control specimens, we previously ignored the real problem that both healthy and CBD bone marrow mononuclear cells are a mixture of different categories of cells including monocytes, lymphocytes, hematopoietic stem cells, and progenitor cells. Hence, the comparison we performed, in fact, is to identify the differential expression profiles of leukemic B-cell precursors versus a heterogeneous population of cells. To some extent, CBD in our study represented the average lncRNA and mRNAs expression profiles of mononuclear cell system.

In addition, trans-patterns were used to predict the co-expression network of lncRNAs with mRNAs, unlike the guilt–by–association approach of the aforementioned previous study. The present study also obtained more high thresholds of FDR (< 0.01) and high Pearson correlation scores (0.99). Here, five novel lncRNAs were identified, which interacted with at least seven mRNAs, including FAT1, SOX11, and EYA4. The functional roles of these lncRNAs remain unknown. Nevertheless, according to the alternative mRNAs in coexpression networks, these lncRNAs may exert exclusive characters by regulating downstream mRNAs. Among them, FAT1 was highly expressed in a large proportion of cases of T-ALL and B-ALL was implicated in Wnt signaling and hippo signaling. FAT1 was also found to cooperate with NOTCH in driving T-ALL in vivo; this suggests that it might be a potential biomarker in carcinogenic roles [37, 38]. Moreover, previous studies have shown SOX11 to be a useful diagnostic marker for mantle cell lymphoma [39]. It has been reported that the high expression of SOX11 leads to alterations of gene expression that are typically associated with cell adhesion, migration, and differentiation. Furthermore, its expression marks a group of patients with good outcomes [40]. Furthermore, Huang et al. demonstrated that AML1-ETO-fused protein triggers the epigenetic silencing of the EYA4 gene, contributing to leukemogenesis in t (8;21) AML. This suggests that the EYA4 gene might be a novel therapeutic target for AML. In addition, although here the co–expression lncRNA–mRNA pairs were captured from RNA–Seq data, it is well established that co–expression correlation does not imply causation. Thus, gene perturbation experimental data would be necessary to gain insight into the possible regulatory relationships, or to infer causal gene co–expression patterns.

## Conclusions

In conclusion, the present study identified differentially expressed lncRNAs and mRNAs between CBD and B-ALL, via NGS. Some of these genes were randomly selected and assessed by qRT-PCR in vitro. Go and KEGG analyses were performed, and the obtained co-expression network indicated that the lncRNAs MSTRG.27994.3, MSTRG.21740.1, ENST00000456341, MSTRG.14224.1, and MSTRG.20153.1 may have bioactive effects in B-ALL. Therefore, the present study may provide pathological sagacity into the mechanisms underlying B-ALL. More importantly, these results provide a basis for further studies into the function and mechanism of special mRNAs and lncRNAs in B-ALL. However, further functional investigations are required to confirm these results.

## Supplementary Information

**Additional file 1: Table S1.** The main clinical and laboratory features of all included samples in the study. **Table S2.** All significantly upregulated and downregulated long non-coding RNAs. **Table S3.** All significantly upregulated and downregulated mRNAs. **Table S4.** The primers used by RT-qPCR. **Table S5.** Correlation of co-expression network between lncRNA and mRNA. **Table S6.** Summary of related work on lncRNAs in B-ALL.

**Additional file 2: Figure S1.** Flow chart of bioinformatics analysis, with the content of rectangular box representing the analysis performed and blue words indicating the software used. **Figure S2.** Identification of differentially expressed long non-coding RNAs (lncRNAs) in B-ALL. (A) Venn diagram presents overlapping relationships, and the numbers indicate novel lncRNA counts. (B) Types and counts of different lncRNAs classified into six categories according to the genomic loci of their neighboring genes. **Figure S3.** Disease ontology (DO) and Reactome analysis showed the top 20 enriched terms of differentially expressed mRNAs in B-ALL. (A) DO Enrichment analysis (B) Reactome Enrichment analysis in B-ALL compared with CBD groups. **Figure S4.** GO and pathway analyses of mRNAs associated with differentially expressed lncRNAs by trans-pattern. (A) Biological process category. (B) Cellular component category. (C) Molecular function category. (d) Canonical signaling pathways.

## Data Availability

All data analyzed during this study are included in this article.

## Abbreviations

CLL: Chronic lymphocytic leukemia
B-ALL: B cell acute lymphoblastic leukemia
lncRNA: Long non-coding RNA
CBD: Common blood diseases
DETs: Differentially expressed transcripts
GO: Gene Ontology
KEGG: Kyoto Encyclopedia of Genes
FC: Fold change
