# Supplementary material for: Differential mRNA and long noncoding RNA expression profiles in pediatric B-cell acute lymphoblastic leukemia patients
Source: BMC Pediatr. 2022 Jan 3;22:10. doi: 10.1186/s12887-021-03073-5 (PMC8722040; doi:10.1186/s12887-021-03073-5)
Supplement: Supplementary file 2 — Additional file 2: Figure S1. Flow chart of bioinformatics analysis, with the content of rectangular box representing the analysis performed and blue words indicating the software used. Figure S2. Identification of differentially expressed long non-coding RNAs (lncRNAs) in B-ALL. (A) Venn diagram presents overlapping relationships, and the numbers indicate novel lncRNA counts. (B) Types and counts of different lncRNAs classified into six categories according to the genomic loci of their neighboring genes. Figure S3. Disease ontology (DO) and Reactome analysis showed the top 20 enriched terms of differentially expressed mRNAs in B-ALL. (A) DO Enrichment analysis (B) Reactome Enrichment analysis in B-ALL compared with CBD groups. Figure S4. GO and pathway analyses of mRNAs associated with differentially expressed lncRNAs by trans-pattern. (A) Biological process category. (B) Cellular component category. (C) Molecular function category. (d) Canonical signaling pathways. [file 12887_2021_3073_MOESM2_ESM.docx]

Figure S1


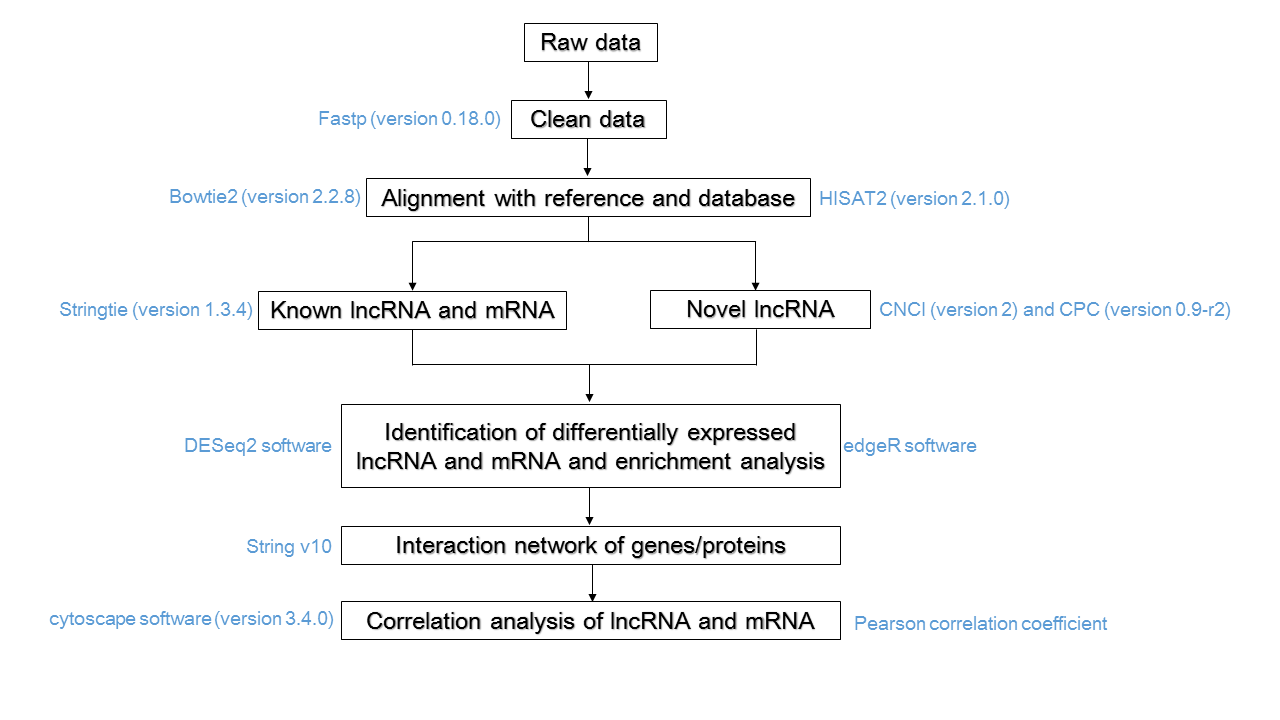


**Figure S1** Flow chart of bioinformatics analysis, with the content of rectangular box representing the analysis performed and blue words indicating the software used.


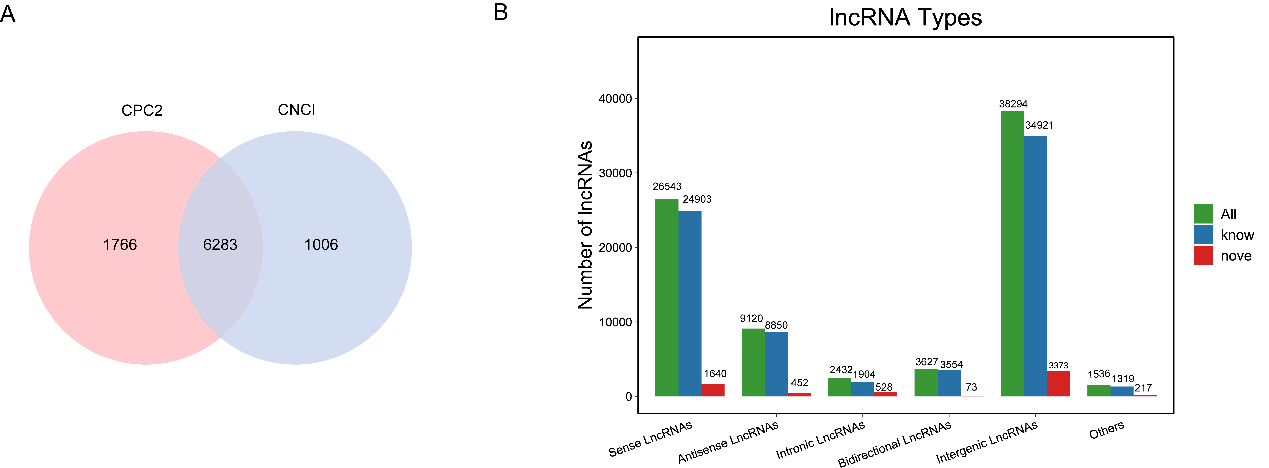
Figure S2

**Figure S2** Identification of differentially expressed long non-coding RNAs (lncRNAs) in B-ALL. (A) Venn diagram presents overlapping relationships, and the numbers indicate novel lncRNA counts. (B) Types and counts of different lncRNAs classified into six categories according to the genomic loci of their neighboring genes.

Figure S3


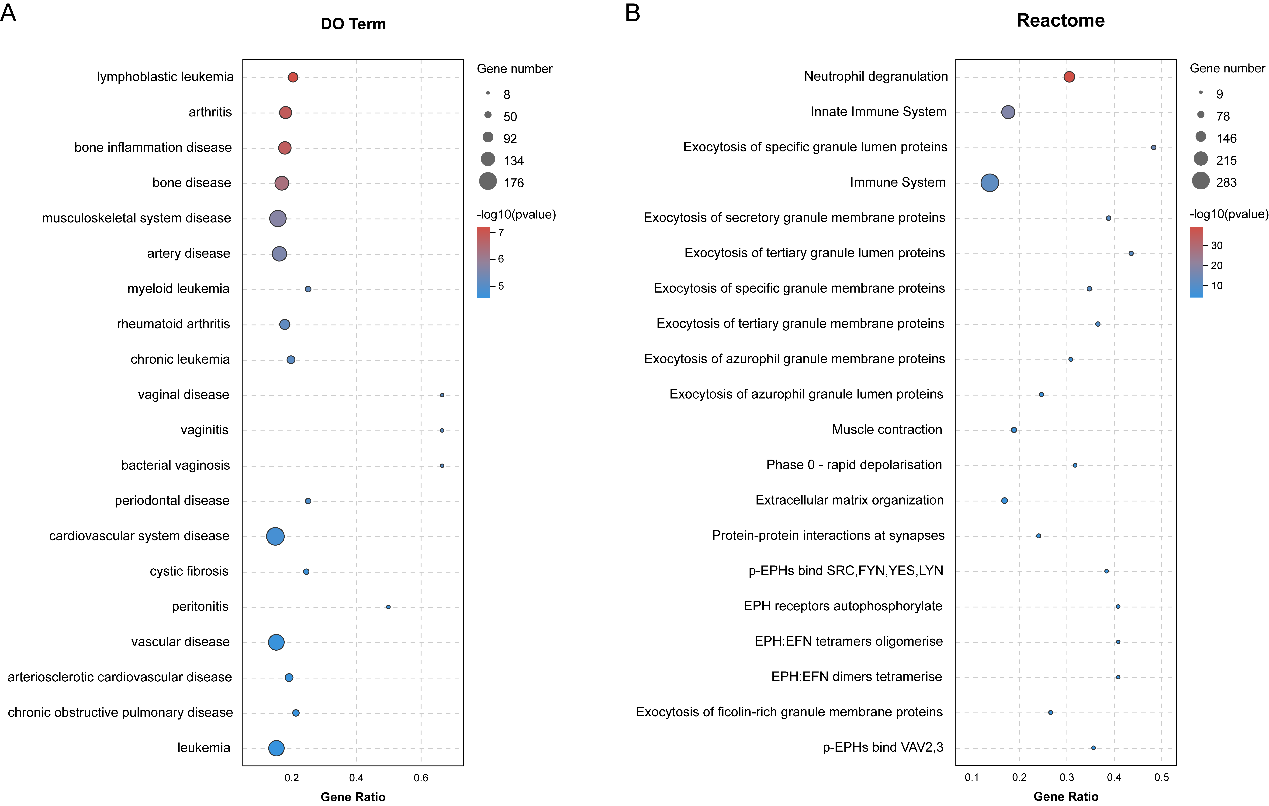


**Figure S3** Disease ontology (DO) and Reactome analysis showed the top 20 enriched terms of differentially expressed mRNAs in B-ALL. (A) DO Enrichment analysis (B) Reactome Enrichment analysis in B-ALL compared with CBD groups.

Figure S4


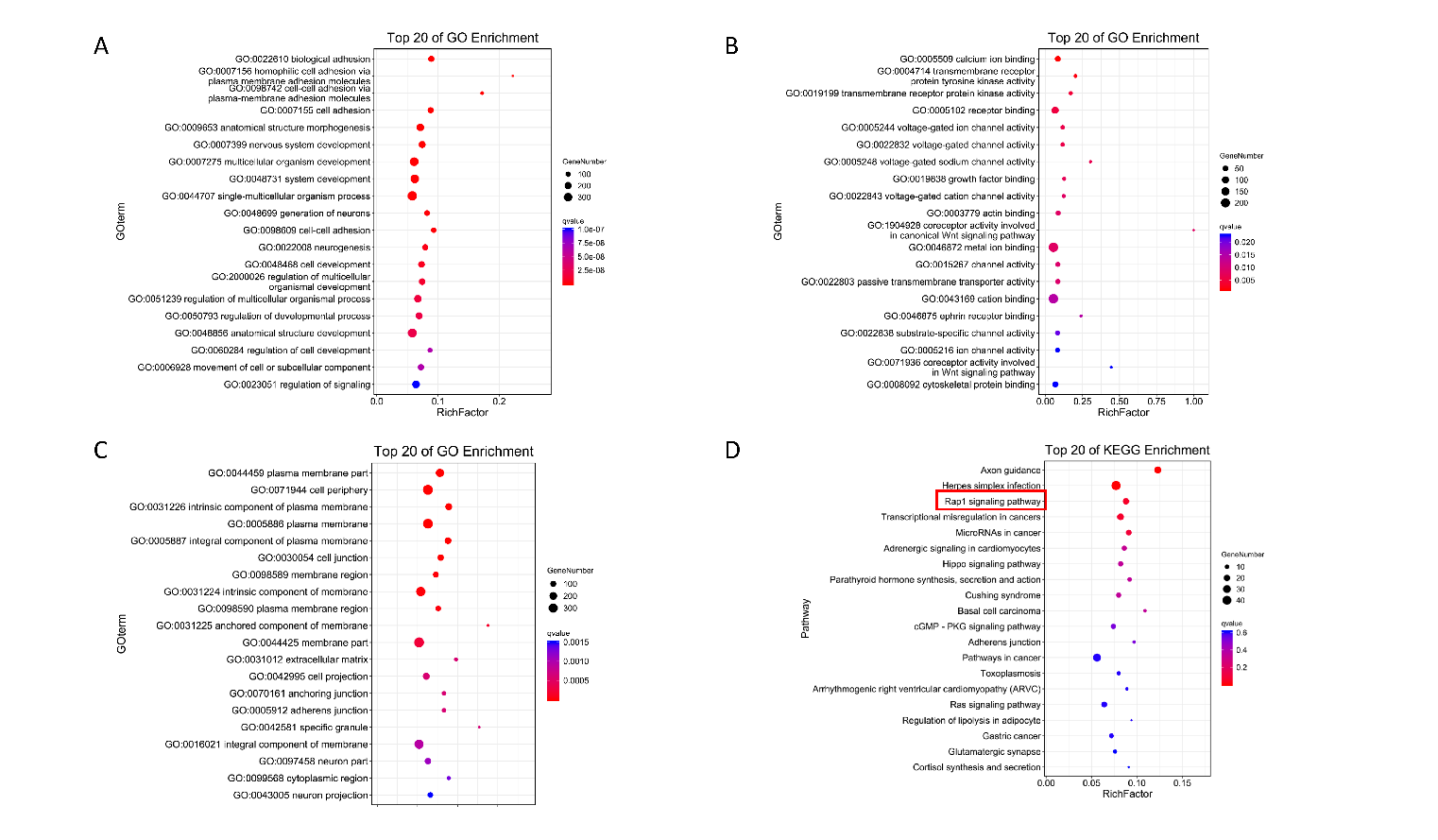


**Figure S4** GO and pathway analyses of mRNAs associated with differentially expressed lncRNAs by trans-pattern. (A) Biological process category. (B) Cellular component category. (C) Molecular function category. (d) Canonical signaling pathways.
